# Nitrogen Supply Affects Yield and Grain Filling of Maize by Regulating Starch Metabolizing Enzyme Activities and Endogenous Hormone Contents

**DOI:** 10.3389/fpls.2021.798119

**Published:** 2022-02-02

**Authors:** Kai Yue, Lingling Li, Junhong Xie, Yaoquan Liu, Jianhui Xie, Sumera Anwar, Setor Kwami Fudjoe

**Affiliations:** ^1^State Key Laboratory of Aridland Crop Science, Gansu Agricultural University, Lanzhou, China; ^2^College of Agronomy, Gansu Agricultural University, Lanzhou, China; ^3^Institute of Crop Science, Xinjiang Academy of Agri-Reclamation Sciences, Shihezi, China; ^4^Institute of Molecular Biology and Biotechnology, The University of Lahore, Lahore, Pakistan

**Keywords:** nitrogen application, maize yield, hormone, starch metabolizing enzymes, grain filling

## Abstract

This study aimed to examine the effect of nitrogen (N) application rate and time on yield, grain filling, starch metabolizing enzymes, and hormones of maize based on a long-term field experiment initiated in 2012. The total N fertilizer dose [(0 (N0), 100 (N1), 200 (N2), and 300 (N3) kg N ha^–1^] was split into two (T1, one-third at sowing and two-thirds at the six-leaf stage) or three (T2, one-third each at sowing, six-leaf, and eleven-leaf stage) times application. The results showed that the highest yield was obtained under N3T2, N2T1, and N3T2 in 2018, 2019, and 2020, which was 222.49, 185.31, and 194.00% than that of N0 in each year, respectively. N2 and N3 significantly increased the yield through enhancing ears ha^–1^, grains per plant, and 100-grain weight; however, N2 and N3 did not show a significant difference in yield and above-yield components. In addition, N application time did not significantly change yield under the same N rate. N0 limited the activities of starch metabolizing enzymes, resulting in insufficient accumulation of sucrose and starch. The contents of indole-3-acetic acid, cytokinin, abscisic acid, and gibberellin were decreased under N0 during grain filling. The average grain-filling rate and maximum grain-filling rate (*G*_max_) and grain weight increment achieving *G*_max_ increased under N2 and N3, and the grain-filling parameters were positively correlated with 100-grain weight. In conclusion, 200 kg N ha^–1^ with one-third application at sowing and two-thirds application at the six-leaf stage is a suitable N supply way to improve starch metabolizing enzymes, regulate hormone content, and enhance grain-filling rates, and thus increasing the maize yield in the semiarid Loess Plateau of China.

## Introduction

Nitrogen (N) is a major nutrient for maize (*Zea mays* L.) production and also takes part in the plant physiological process as a significant component, such as enzymes, hormones, and amino acids (Thind et al., 2011; Ciampitti and Vyn, 2012). Therefore, to get a higher yield, high N application is the most common practice for farmers in the semiarid Loess Plateau of China. However, excessive N leads to abundant losses of N fertilizer as well as restraining antioxidant enzymes of cereal leaf and decreasing grain yields (Kong et al., 2017). In addition, the suitable time of N applied could increase maize yield, but the variation of the yield is tightly associated with the N rate and external environment (Ning et al., 2017; Banger et al., 2018). These studies suggest that excessive high N rate and inappropriate N application time could decrease crop yield. However, the influence of N rate and time on maize yield is vague in the semiarid Loess Plateau of China. Grain weight, ears ha^–1^, and grains per ear are the decisive factor of yield formation (Lu et al., 2017). Previous studies showed that N fertilization significantly increased grain yield by enhancing the grain weight, ears ha^–1^, and grains per ear (Srivastava et al., 2018; Wei et al., 2019). The appropriate N enhances grain weight as a result of increasing effective grain-filling duration and rate (Wei et al., 2019). However, the heavy use of N results in slow grain filling and unfavorably delayed senescence which prolongs grain-filling duration, leading to low grain weight (Yang and Zhang, 2006). So, it is vital to clarify the physiological mechanisms of grain filling for the increase of maize yield under different N application rates and times in the semiarid Loess Plateau of China.

The grain-filling process could be divided into gradual increase period (GIP), rapid increase period (RIP), and slight increase period (SIP) through the generative parameters (Wei et al., 2019). Cao et al. (2015) found that appropriate N application increased active grain-filling period (AGP) and maximum grain-filling rate (*G*_max_), which parameters calculated by logistic equation, and the variation of yield may due to AGP and *G*_max_, which were positively correlated with yield. Wang et al. (2018) found that *G*_max_ and AGP increased significantly under controlled-release urea, so N application time also affects grain yield by affecting grain filling. For some large ear crops, insufficient grain filling of grains is one of the main factors limiting yield potential (Yang and Zhang, 2010). Thus, a key approach to increasing grain weight is to maintain a good grain-filling process. However, there has been little agreement on what N rate and time affect grain filling in maize in the semiarid Loess Plateau of China.

As momentous substances regulating grain filling, starch-metabolizing enzymes and endogenous hormones contribute significantly to grain development (Fahy et al., 2018; Wang and Zhang, 2020). The concerted activities of enzymes such as ADP-glucose pyrophosphorylase (AGPase) and starch synthases (SSs) lead to the synthesis of the starch (Thitisaksakul et al., 2012). Starch is the principal component of maize grain that determines grain weight (Cazetta et al., 1999), and N-limitation decreases grain starch accumulation by affecting carbohydrate biosynthetic enzyme activity (Seebauer et al., 2010). Furthermore, sucrose synthase (SuSy) and sucrose phosphate synthase (SPS) can regulate starch accumulation by affecting sucrose synthesis (Ning et al., 2018). Studies reported that AGPase and SSs activities increased with the increase of N application, which indicated these two enzymes might play a more critical role in the biosynthesis and regulation of the accumulation rate of corn starch (Liu et al., 2021). In addition, AGPase and SSs activities in grains could be improved by proper N application time, and different enzymes act differently at different grain-filling periods (Li et al., 2005). The endogenous hormones in grains such as indole-3-acetic acid (IAA), cytokinin (CTK), abscisic acid (ABA), and gibberellin (GA) are involved in the regulation of grain-filling rate and duration (Liu et al., 2013; Xu et al., 2013). The reciprocal regulation between hormones and enzymes and the balance of hormones play a crucial role in the final grain weight (Fu et al., 2013). ABA content regulates the signaling pathway of sugar synthesis, and several studies have documented an increase in ABA content by N application compared to non-N-fertilized (Rook et al., 2001; Qin et al., 2013). The proper N application time is conducive to increasing CTK content in grains, promoting the accumulation of dry matter into the grains, and increasing grain weight (Luo et al., 2018). Jiang et al. (2009) reported that high GA content contributes to maize grain-filling rate in the grain-filling stage.

It has been proved that starch-metabolizing enzymes and endogenous hormones regulate starch accumulation and grain filling. However, the effects of the N rate and time on yield and grain filling *via* regulating enzymes and hormones in grain filling were unclear, especially in the Loess Plateau under field conditions. Therefore, we analyze the variation in maize yield, yield components, grain-filling parameters, starch synthase activities, and endogenous hormone content under different N rates and times in this study. The objective of this study was to: (i) identify the contribution of grain filling and yield components to yield under different N applications, (ii) investigate the effect of N fertilizer on starch accumulation through regulating starch-metabolizing activities, and (iii) confirm the relationship between hormone contents and grain-filling characteristics, which determined by logistic and standardized analytical modeling. The research may provide a theoretical basis for optimizing N application for maize in the semiarid Loess Plateau of China.

## Materials and Methods

### Site Description

**FIGURE 1:**
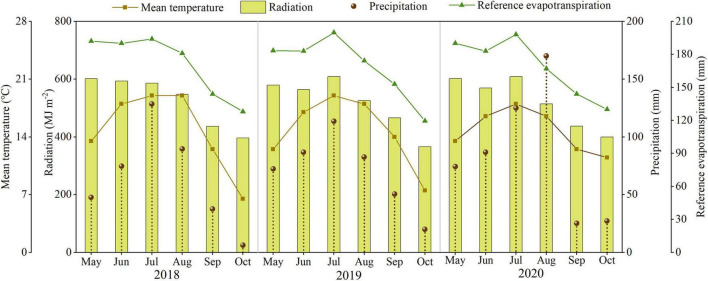
The precipitation, mean temperature, cumulative radiation, and evapotranspiration during the maize growth period in 2018, 2019, and 2020.

The field study was conducted at the Dingxi Experimental Station (35°28′N, 104°44′E) of the Gansu Agricultural University, Gansu Province, Northwest China. The area is a semiarid Loess Plateau with an average altitude of 2,000 m. The experimental data for 2018, 2019, and 2020 have been presented in this study. The aeolian soil of the site is prone to erosion and classified as a Calcaric Cambisol (Food and Agriculture Organization [FAO], 1990). The soil at 0–20 cm depth has a sandy loam texture with 50% sand, moderate to low fertility, slightly alkaline pH (8.3), soil organic carbon of 7.65 g kg^–1^, available potassium of 220 mg kg^–1^, and Olsen’s phosphorus of 13 mg kg^–1^, and the soil mineral N status before the sowing as shown in Table 1. Annual frost-free period of 140 days, and annual radiation is 5,930 MJ m^–2^. The average annual growing season precipitation, mean temperature, cumulative radiation, and evapotranspiration at the site during 2018, 2019, and 2020 are shown in Figure 1. In the crop-growing season, precipitation (May–September) was 377.7, 405.3, and 481.0 mm in 2018, 2019, and 2020, respectively. Total sunny days in the main grain-filling period (August and September) were 13, 25, and 21 days in 2018, 2019, and 2020, respectively, according to meet record 2018 was a year with the lowest precipitation and the least sunny days during grain filling.

**TABLE 1:** The soil mineral N status (mg kg^–1^) before sowing.

Year	N0	N1T1	N1T2	N2T1	N2T2	N3T1	N3T2
2018	8.06	11.79	9.79	19.03	20.83	21.65	21.36
2019	8.47	11.51	10.24	21.85	21.10	23.75	22.87
2020	8.17	9.31	9.09	21.51	21.04	23.51	22.96

*N0, no N fertilizer; N1, 100 kg N ha^–1^; N2, 200 kg N ha^–1^; N3, 300 kg N ha^–1^. T1, one-third of the full N rate corresponding to the treatment at sowing and the remaining two-thirds at V6; T2, one-third of the full N rate corresponding to the treatment at sowing, V6, and V11.*

### Experiment Design

The four N fertilization levels were divided into two and three application times in a completely randomized block design and three replications per treatment. The four N fertilization levels were: N0 (no N fertilizer), N1 (100 kg N ha^–1^), N2 (200 kg N ha^–1^), and N3 (300 kg N ha^–1^). For the first N application time (T1), one-third of the total N rate was applied at sowing while the remaining two-thirds were at the six-leaf collar stage (V6). In the second N application time (T2), one-third of the total N rate was applied at sowing and V6 and eleven-leaf collar (V11) stages. N fertilizer was applied using urea (46% N) and phosphorus (150 kg ha^–1^) as P_2_O_5_. No potassium fertilizer was applied as available potassium (220 mg kg^–1^) in the soil is enough for maize production. Completely film mulched alternate narrow and wide ridges with furrow planting, which is a new planting technology, can increase soil temperature and reduce direct evaporative losses. The area of each plot was 18.7 m^2^ (4.4 m × 4.25 m). The alternate wide (0.7 m) and narrow (0.4 m) ridges were mulched with transparent plastic films (1.1 m wide) before sowing maize. The maize, cv. Xianyu 335, which is the most popular variety in this area, were sown in furrows at a target final stand of 52,500 plants ha^–1^ in late April, which is 98 plants per plot. Maize plants were harvested in early October of each year.

### Measurements and Calculations for Grain Filling, Enzymes, and Endogenous Hormones Measurements

In each plot, 24 plants were labeled before pollination. Three ears were selected from 24 labeled plants each week after pollination, that is, at 7, 14, 21, 28, 35, 42, 49, and 56 days after pollination (DAP), respectively. A total of 200 grains were then randomly sampled from the middle part of the three ears (total grains in each ear is approximately 350 grains), counting from the bottom to the top from the tenth to the twentieth row (Fang et al., 2020), and divided into two portions. The first portion of grains (7, 14, 21, 28, and 35 DAP) was frozen in liquid N at once, carried back to the laboratory, and stored in −80°C fridge for starch synthetic enzymes and endogenous hormones measurements. The second portion of grains (7, 14, 21, 28, 35, 42, 49, and 56 DAP) was dried in an oven for 30 min at 105°C and then at 80°C to constant weight, the grains were then weighed, dried grains (7, 14, 21, 28 and 35 DAP) were also sampled to determine the sucrose, starch, and protein content.

#### Grain-Filling Traits

The grain-filling process was modeled using the following logistic equation (Wei et al., 2019):


(1) W=A/(1+Be)-Ct

where *W* is the grain weight, *A*, *B*, and *C* are the equation coefficients, *t* is the days after pollination.

The grain–filling parameters were calculated as Wei et al. (2019):


(2) Timeformaximalgrain-fillingrate(T)max=lnB/C

(3) Grain⁢weight⁢increment⁢achieving⁢maximum⁢grain⁢-⁢fillingrate(W)max=A/2

(4) Maximumgrain-fillingrate(G)max=(C×W)max[1-(W/maxA)]

(5) Active⁢grain⁢-⁢filling⁢period⁢(A⁢G⁢P)=6/C

The duration, weight, and average grain-filling rate of GIP, RIP, and SIP, we set as *T*_1_, *W*_1_, *G*_1_, *T*_2_, *W*_2_, *G*_2_, *T*_3_, *W*_3_, and *G*_3_, the parameters were calculated as Wei et al. (2019).


(6) Averagegrain-fillingrate(G)max=W/56

Where *W* is the 100-grain weight at 56 DAP.

#### Starch and Sugar Analysis

The grain starch content (%) was determined using the anthrone-sulfuric acid method (Zou, 1995). First, 0.2 g of grain powder was weighed, 7 ml of 80% ethanol was added into 80°C water bath for 30 min, centrifuged at 4,000 rpm for 5 min, the precipitate is retained, and the extraction is performed three times. Then, 2 ml of deionized water was added to the precipitate and the ethanol was evaporated at 80°C and gelatinized in a boiling water bath for 15 min. After cooling, 2 ml of 9.2 M HClO_4_ was added and hydrolyzed for 15 min, and then 4 ml of deionized water was added and centrifuged for 10 min. Subsequently, the supernatant was poured into a volumetric flask. Then, 2 ml of 4.6 M HClO_4_ was added to the precipitate and 6 ml of deionized water was added after 15 min of extraction, centrifuged for 10 min, and diluted to 50 ml with deionized water. Furthermore, 0.5 ml of 2% anthrone and 5 ml of concentrated sulfuric acid were added to the supernatant. The starch content was calculated according to the absorbance measured at 620 nm.

The grain sucrose content (mg g^–1^) was determined using the resorcinol method (Zhang and Qu, 2003). First, 0.2 g sample was weighed, 4 ml of 80% ethanol extract was added into 80°C water bath for 40 min and centrifuged, the supernatant was retained, the precipitate was extracted two times with 2 ml of 80% ethanol and diluted to 10 ml. Then, 0.4 ml of the supernatant was taken, added to 200 μl of 2 M NaOH, mixed thoroughly, and subsequently boiled in a water bath for 10 min. Then, 2.8 ml of 30% HCl was added and mixed well and kept at 80°C for 10 min. At last, 1 ml 1% resorcinol was added to the mixture, mixed thoroughly, and kept at 80°C for 10 min again. After cooling, the absorbance at 480 nm was measured to calculate sucrose content.

#### Sucrose Synthase and Sucrose Phosphate Synthase

The grains samples (0.5 g) were homogenized with the extraction buffer containing 5 ml of 50 mM HEPES-NaOH (pH 7.5) in ice-chilled (0°C∼4°C) mortar. The mixture was centrifuged at 10,000 × *g* for 10 min, and the supernatant was used for enzyme determination (Yang et al., 2018).

Sucrose synthase (SuSy) assay was performed by using the proposed method with modifications (Yang et al., 2018). About 50 μl HEPES-NaOH (pH 7.5), 20 μl 100 mM UDPG, 20 μl 100 mM fructose, and 20 μl 50 mM MgCl_2_ were added to 50 μl enzyme solution, and then the reaction was performed at a temperature of 30°C for 30 min. Later, 0.2 ml of 2 M NaOH was added, the reaction was terminated, and then boiling water was used to heat the solution for 10 min. After that, 2 ml 30% HCl was poured into it and remained at a temperature of 80°C for 10 min. Then, 1 ml 1% resorcinol was poured into it and remained for about 10 min. Finally, it was cooled down, and 3.64 ml of deionized water was added. Later, at 480 nm, optical density was obtained, and the amount of sucrose produced was determined by the unit activity. It could be concluded that the SPS determination process was equal to SuSy, except the fructose has been replaced by fructose-6-phosphate (Yang et al., 2018).

#### Assay of Starch Synthesis Enzyme

Starch synthases (SSS) and AGPase were determined using the ELISA kit that was supplied by Jiangsu Meibiao Biotechnology Co., Ltd., (Yancheng, China).

#### Endogenous Hormone

According to previous studies (Yang et al., 2001; Liang et al., 2017), the endogenous ABA, IAA, GA, and CTK were determined by enzyme-linked immunosorbent assay (ELISA), the ELISA kits were supplied by Jiangsu Meibiao Biotechnology Co., Ltd., (Yancheng, China).

### Yield and Yield Components

At the physiological maturity stage, 10 plants were randomly selected to determine ears ha^–1^, grains per plant, and 100-grain weight. As for grain yield, the sampling area was excluded, then yield per hectare was calculated based on the actual harvest yield from each plot.

### Statistical Analysis

We used ANOVA to analyze data with SPSS 25.0 (SPSS Institute Incorporation, Armonk, NY, United States). Treatments were compared using Duncan’s test at the 0.05 probability level. Pearson correlation analysis and canonical correlation analysis were also performed with the help of SPSS 25.0. Figures were made with Origin 2019.

## Results

### Grain Yield and Yield Components

**FIGURE 2:**
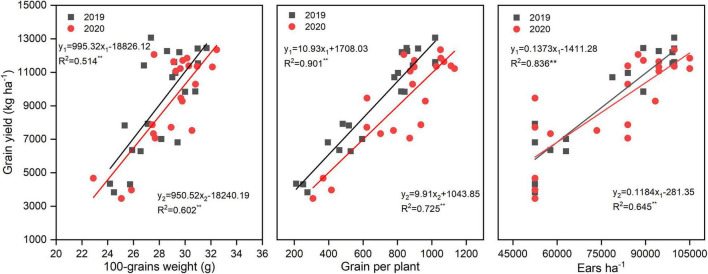
Correlations between yield, 100-grains weight, grains per plant, and ears ha^–1^. y_1_ and y_2_ represent and 2020 simulation equation, respectively.

N rate significantly altered the yield and yield components; however, N application time and N rate and time interaction did not significantly affect the yield and yield components (Table 2). There was a huge difference between years, the annual average yield among all the treatments was 6,578, 9,188, and 9,265 kg ha^–1^ in 2018, 2019, and 2020, respectively. The highest yield was obtained under N3T2, N2T1, and N3T2 in 2018, 2019, and 2020, which was 222.49, 185.31, and 194.00% than that of N0 in each year, respectively. Furthermore, the difference in yield is not significant under N2 and N3 (except for N2T2 in 2018). Under the N2 and N3, ears ha^–1^, grains per plant, and 100-grain weight were enhanced compared to N0, but no significant differences were shown between N2 and N3. Under N0 treatment, there were 52,500 ears ha^–1^ (except 53,375 ears ha^–1^ in 2018), however, it cumulated up to 87,500–99,750 ears ha^–1^ under N2 and N3. Simultaneously, compared with N0, grains per plant of N2 and N3 increased by 240.21 and 280.47% in 2019, and 163.63 and 176.03%. No significant difference was found between N1, N2, and N3 in the 100-grain weight, N2 and N3 significantly increased by 19.19% and 18.88% in 2019, and 19.41% and 28.87%. In addition, the yield of maize was positively correlated with 100-grain weight, grains per plant, and ears ha^–1^ (Figure 2). The most relevant is the grains per plant, followed by ears ha^–1^, and 100-grain weight.

**TABLE 2:** Effects of different N rates and application times on grain yield and yield components of maize.

N	*T*	Grain yield (kg ha^–1^)			Ears ha^–1^			Grains per plant		100-grains weight (g)	
		2018	2019	2020	2018	2019	2020	2019	2020	2019	2020
N0		2,649d	4,156c	4,032c	53,375d	52,500b	52,500c	246c	365d	24.81b	24.60b
N1	T1	6,572bc	6,850b	8,345b	73,500c	57,750b	63,000bc	491b	727c	26.52ab	28.69a
	T2	5,708c	7,214b	7,309b	68,250c	56,000b	71,750b	503b	783bc	27.63ab	28.59a
N2	T1	7,835ab	11,859a	10,543a	99,750a	94,306a	88,278ab	850a	896abc	29.93a	29.40a
	T2	6,795bc	10,935a	11,309a	87,500b	87,500a	98,000a	827a	1,026a	29.21a	29.35a
N3	T1	7,941ab	11,517a	11,461a	99,750a	99,556a	94,500a	977a	941ab	28.99a	30.96a
	T2	8,543a	11,786a	11,853a	98,000a	92,750a	96,250a	898a	1,071a	30.00a	30.97a
N		**	**	**	**	**	**	**	**	*	*
T		ns	ns	ns	ns	ns	ns	ns	ns	ns	ns
N*T		ns	ns	ns	ns	ns	ns	ns	ns	ns	ns

*Different letters indicate significant differences at p < 0.05 between different N applications within same year.*

**Significant differences at p < 0.01; *significant differences at p < 0.05; ns indicates non-significant difference.

### Sucrose and Starch Contents

**FIGURE 3:**
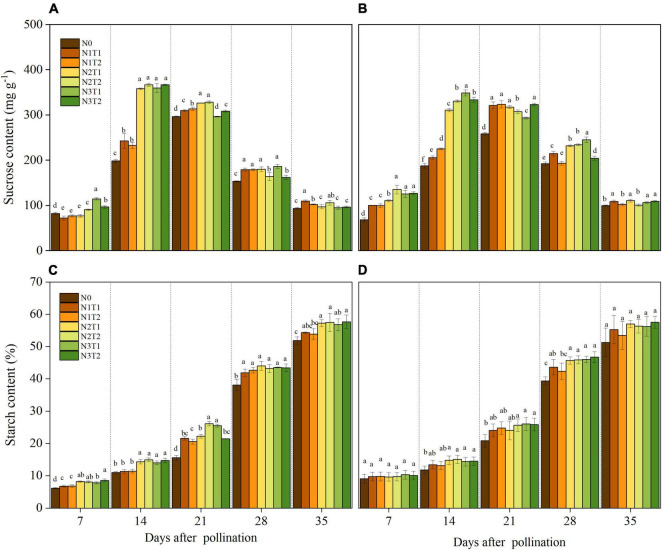
Effects of N rate and time on the content of sucrose and starch in 2019 **(A,C)** and 2020 **(B,D)**. N rate, N0, no N fertilizer; N1, 100 kg N ha^–1^; N2, 200 kg N ha^–1^; N3, 300 kg N ha^–1^. N application time, T1, one-third of the full N rate corresponding to the treatment at sowing and the remaining two-thirds at V6; T2, one-third of the full N rate corresponding to the treatment at sowing, V6, and V11. Different letters indicate significant differences at *p* < 0.05.

Within 7–35 DAP, the sucrose content in grains was affected by the N rate (Figures 3A,B). The highest sucrose content was recorded under N2 and N3 treatments at the 14 DAP except for N2T1 in 2020. Under N0 and N1, the maximum sucrose content appeared at 21 DAP and then decreased with the growth stage. The sucrose content for N2 and N3 was dramatically higher than that for N0 at 14 DAP, and no significance was observed between N2 and N3 for sucrose content. Under the same N rate, N application time did not affect sucrose content significantly.

The starch content increased consistently with time after pollination (Figures 3C,D). After 21 DAP, the starch content under N2 and N3 was increased as compared to N0. There was no significant difference in starch content between T1 and T2 under the same N rate. On average, compared with N0, N2, and N3 increased by 10.62, 10.35, and 10.44, and 10.86% at 35 DAP in 2019 and 2020, respectively.

### Sucrose Synthase and Sucrose Phosphate Synthase Activities

**FIGURE 4:**
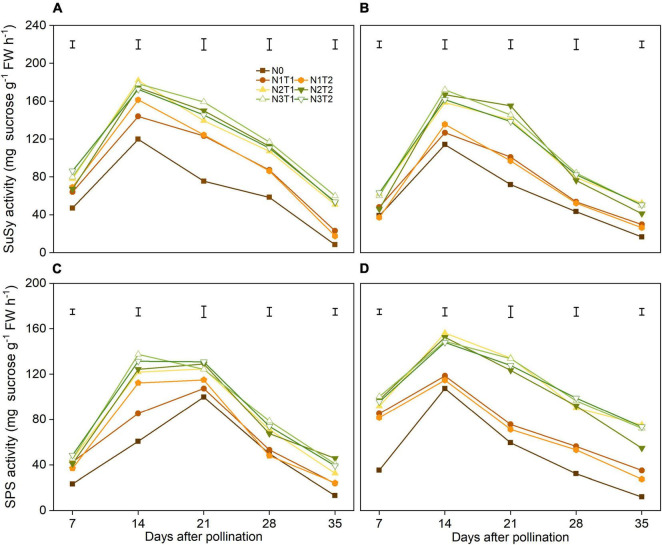
Effects of N rate and time on the activities of SuSy and SPS in 2019 **(A,C)** and 2020 **(B,D)**. SuSy, sucrose synthase; SPS, sucrose phosphate synthase. The vertical bars represent the LSD (*n* = 3).

There was no significant difference in N2 and N3 of SuSy activities, and the variation of SuSy activities was not significant in T1 and T2 under the same N rate (Figures 4A,B). During grain filling, N2 and N3 increased SuSy activity significantly, and the SuSy activities reached a peak at 14 DAP. On average, N2 increased the SuSy activity at 14 DAP by 48.6% and 42.5% compared with N0 in 2019 and 2020, respectively.

Nitrogen affected the SPS activity, however, the peak of SPS was different in 2 years (Figures 4C,D). The N2 and N3 had significantly increased the SPS activities from 7 DAP and 35 DAP compared with N0. In general, N application time did not alter SPS activities significantly under the same N rate.

### ADP-Glucose Pyrophosphorylase and Starch Synthases Activities

**FIGURE 5:**
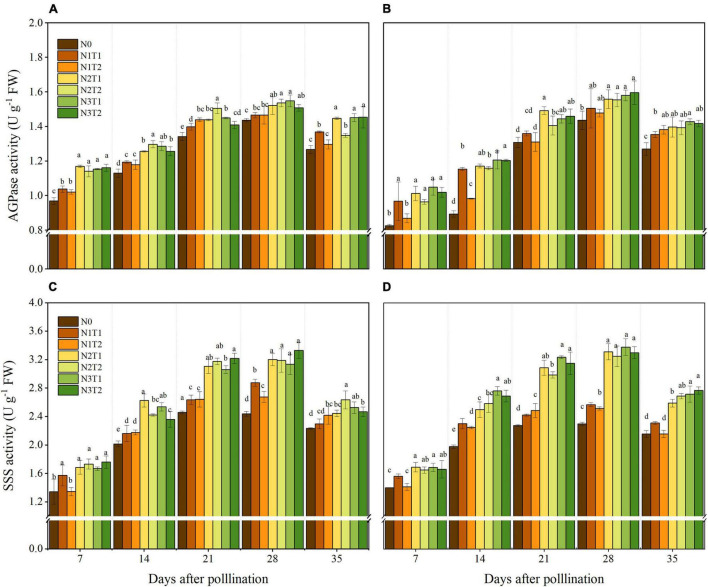
Effects of N rate and time on the activities of AGPase and SSS in 2019 **(A,C)** and 2020 **(B,D)**. AGPase, ADP-glucose pyrophosphorylase, starch synthases. Different letters indicate significant differences at *p* < 0.05.

The activities of AGPase and SSS showed variation with the N supply and peaked at 28 DAP (Figure 5). In the grain-filling period, AGPase activity first increased and then decreased, and N2 and N3 increased AGPase activity compared to N0. No significant differences were obtained between N2 and N3 at 28 DAP, and N2 increased the AGPase by 8.37 and 6.39% in 2019 and 2020 compared with N0, respectively. The AGPase activities for different N application times were not significantly different under different N rates. Similarly, N application time also did not alter SSS activities significantly. Within 7–35 DAP, N2 and N3 increased SSS activities compared to N0, and N2 and N3 did not show a significant difference. At the 28 DAP, N2 significantly increased the SSS activities by 31 and 42.8% compared with N0 in 2019 and 2020.

### Grain-Filling Characteristic Parameters

Progress in grain filling was well-simulated by the logistic model under different N rates and time applications (*R*^2^ ≥ 0.988) (Table 3). The *G*_max_, *W*_max_, *G*_max_, and the AGP increased from N0 to N2, but N2 and N3 do not have a noticeable difference. Different N times do not show significant effects on maize grain-filling characteristics parameters. N0 and N1T2 prolonged *T*_max_ but reduced *G*_max_, *G*_max_, *G*_max_, and *AGP*. On average, the *G*_max_ was increased by N2 and N3 in both years.

**TABLE 3:** Effects of N rate and time on grain filling characteristics parameters of maize grains.

Year	*N*	*T*	*A*	*B*	*C*	*R* ^2^	*T*_max_	*W*_max_	*G*_max_	*G*_ave_	AGP
2019	N0		20.96c	145.51a	0.135a	0.997	36.89a	10.48c	0.71d	0.34c	44.44a
	N1	T1	25.29b	94.92bc	0.130a	0.988	35.02ab	12.64b	0.82c	0.42b	46.15a
		T2	25.39b	111.87ab	0.132a	0.990	35.74ab	12.70b	0.84bc	0.41b	45.45a
	N2	T1	29.13a	79.06cd	0.130a	0.999	33.62b	14.57a	0.95a	0.49a	46.15a
		T2	28.29a	73.32cd	0.129a	0.999	33.29b	14.14a	0.91ab	0.47a	46.51a
	N3	T1	28.95a	56.25d	0.120a	0.999	33.58b	14.48a	0.87bc	0.48a	50.00a
		T2	29.29a	64.15cd	0.124a	0.998	33.56b	14.65a	0.91ab	0.48a	48.39a
	N		**	**	ns		**	**	**	**	**
	T		ns	ns	ns		ns	ns	ns	ns	ns
	N*T		ns	ns	ns		ns	ns	ns	ns	ns
2020	N0		20.47c	86.06a	0.129a	0.993	34.59a	10.24c	0.66d	0.34c	46.76b
	N1	T1	25.67b	51.12b	0.113ab	0.992	34.80a	12.83b	0.72c	0.41b	53.65ab
		T2	25.80b	58.95b	0.116ab	0.994	35.20a	12.90b	0.74bc	0.41b	52.15ab
	N2	T1	29.52a	48.65b	0.113ab	0.993	34.30a	14.76a	0.84a	0.48a	53.00ab
		T2	28.54ab	45.59b	0.112ab	0.991	33.95a	14.27ab	0.80ab	0.47a	53.41ab
	N3	T1	28.66ab	41.45b	0.113ab	0.989	33.14a	14.33ab	0.81ab	0.47a	53.47ab
		T2	31.03a	40.64b	0.106b	0.986	34.94a	15.51a	0.82a	0.49a	56.95a
	N		**	**	*		ns	**	**	**	*
	T		ns	ns	ns		ns	ns	ns	ns	ns
	N*T		ns	ns	ns		ns	ns	ns	ns	ns

*A, B, C represent equation coefficients; R^2^, correlation coefficients.*

*T_max_, the day reaching the maximum grain filling rate; W_max_, grain weight increment achieving maximum grain filling rate; G_max_, maximum grain filling rate; G_max_, average grain-filling rate; AGP, active grain-filling period.*

**Significant differences at p < 0.01; *significant differences at p < 0.05; ns indicates non-significant difference. Different letters indicate significant differences at p < 0.05.

N application time did not significantly affect the duration, weight, and grain-filling rate during GIP, RIP, and SIP under the same N rate (Table 4). Compared with other treatments, N0 and N1 prolonged the duration of GIP but shortened the duration of RIP and SIP. The duration of RIP under N0, N2, and N3 accounted for 27.65, 29.50, and 30.31% in 2019 and 29.16, 31.20, and 31.75% in 2020 of the whole grain-filling duration, respectively. The average grain-filling rate of N2 was 59.6% and 53.8% higher during GIP and 31.4% and 24.6% higher during RIP than N0 in 2019 and 2020, respectively. Similarly, the grain-filling rate of N3 was 65.6 and 61.5% higher during GIP and 25.6 and 23% higher during RIP than N0 in 2019 and 2020, respectively. Correlation analysis showed that 100-grain weight was significantly positively correlated with *W*_max_, *G*_max_, *G*_max_, *G*_1_, *T*_2_, *G*_2_, *T*_3_, and *G*_3_, and negatively correlated with *T*_1_ (Table 5).

**TABLE 4:** Effects of N rate and time on characteristics parameters of the three grain-filling phases of maize grains.

Year	N	*T*	Gradual increase period			Rapid increase period			Slight increase period		
			T_1_	W_1_	G_1_	T_2_	W_2_	G_2_	T_3_	W_3_	G_3_
2019	N0		27.13a	4.43c	0.1632c	19.51a	12.10c	0.6201d	24.28a	4.22c	0.1737d
	N1	T1	24.89b	5.34b	0.2147b	20.26a	14.60b	0.7205c	25.22a	5.09b	0.2019c
		T2	25.76b	5.37b	0.2083b	19.95a	14.66b	0.7347bc	24.83a	5.11b	0.2058bc
	N2	T1	23.49c	6.16a	0.2621a	20.26a	16.82a	0.8301a	25.22a	5.86a	0.2326a
		T2	23.08c	5.98a	0.2589a	20.42a	16.33a	0.7999ab	25.41a	5.69a	0.2241ab
	N3	T1	22.61c	6.12a	0.2706a	21.95a	16.72a	0.7616bc	27.32a	5.83a	0.2134bc
		T2	22.94c	6.19a	0.2699a	21.24a	16.91a	0.7962ab	26.44a	5.90a	0.2231ab
	N		**	**	**	ns	**	**	ns	**	**
	T		ns	ns	ns	ns	ns	ns	ns	ns	ns
	N*T		ns	ns	ns	ns	ns	ns	ns	ns	ns
2020	N0	-	24.33a	4.33c	0.1778d	20.53b	11.82c	0.5767c	25.55b	4.12c	0.1616c
	N1	T2	23.75ab	5.45b	0.2293c	22.90ab	14.89b	0.6527b	28.49ab	5.19b	0.1829b
		T1	23.02ab	5.42b	0.2355c	23.55ab	14.82b	0.6321b	29.31ab	5.17b	0.1771b
	N2	T2	22.23ab	6.03ab	0.2716b	23.45ab	16.48ab	0.7027a	29.18ab	5.75ab	0.1969a
		T1	22.67ab	6.24a	0.2751b	23.27ab	17.04a	0.7324a	28.96ab	5.94a	0.2052a
	N3	T2	22.44ab	6.56a	0.2914a	25.00a	17.91a	0.7173a	31.11a	6.25a	0.2010a
		T1	21.41b	6.06ab	0.2830ab	23.47ab	16.55ab	0.7064a	29.21ab	5.77ab	0.1979a
	N		*	**	**	*	**	**	*	**	**
	T		ns	ns	ns	ns	ns	ns	ns	ns	ns
	N*T		ns	ns	ns	ns	ns	ns	ns	ns	ns

*T_1_, grain-filling duration of gradual increase period; W_1_, increased grain weight of gradual increase period; G_1_, mean grain-filling rate of gradual increase period; T_2_, grain-filling duration of rapid increase period; W_2_, increased grain weight of rapid increase period; G_2_, mean grain-filling rate of rapid increase period; T_3_, grain-filling duration of slight increase period; W_3_, increased grain weight of slight increase period; G_3_, mean grain-filling rate of slight increase period.*

**Significant differences at p < 0.01; *significant differences at p < 0.05; ns indicates non-significant difference. Different letters indicate significant differences at p < 0.05.

**TABLE 5:** Pearson correlation of 100-grain weight and grain-filling parameters of maize grains.

	Year	*T*_max_	*W*_max_	*G*_max_	*G*_max_	AGP	T_1_	G_1_	T_2_	G_2_	T_3_	G_3_
100-grains weight	2019	−0.924**	0.975**	0.973**	0.975**	0.634	−0.904*	0.956**	0.634	0.965**	0.634	0.965**
	2020	−0.427	0.941**	0.870*	0.941**	0.944**	−0.903**	0.959**	0.945**	0.870**	0.944**	0.869**

**Significant differences at p < 0.01; *significant differences at p < 0.05; ns indicates non-significant difference.

### Hormone Content

**FIGURE 6:**
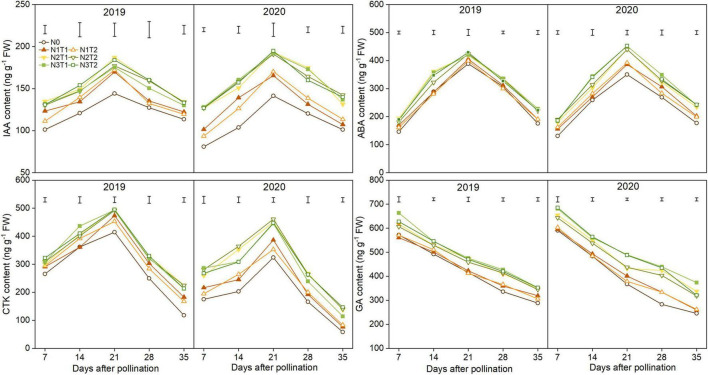
Effects of N rate and time on the content of indole-3-acetic acid (IAA), cytokinin (CTK), abscisic acid (ABA), and gibberellic acid (GA) in 2019 and 2020. The vertical bars represent the LSD (*n* = 3).

The IAA, CTK, and ABA content increased first and then declined; however, GA content declined gradually during the grain-filling process (Figure 6). IAA, ABA, and CTK significantly increased under N2 and N3 compared with N0 and reached a maximum value at 21 DAP. Furthermore, there were no significant differences between N2 and N3, T2 and T1 under the same N rate for most hormones. On average, the peak of the IAA, ABA, and CTK was higher under the N2 than N0 treatments which were increased by 26.2, 8.7, and 19.1% in 2019 and 35.9, 25.6, and 40.7% in 2020. The N2 and N3 treatments significantly improved GA content as compared with N0, while there was no significant difference in GA content between N1 and N0 from 7 to 21 DAP.

### Correlation Between Grain-Filling Characteristic Parameters and Hormone Content

Hormone contents at 7, 14, 28, 35, and 42 DAP were set as variables a_1_, a_2_, a_3_, a_4_, and a_5_, while, *W*_max_, *G*_max_, *G*_max_, *W*_2_, and *G*_2_ were set as b_1_, b_2_, b_3_, b_4_, and b_5_, respectively (Table 6). As Table 6, the variation of grain-filling parameters depended on the content of ABA at 35 DAP and GA at 28 DAP. There were two significant standardized analytical models between IAA content and grain-filling parameters in 2019, and as the first and second typical variables showed that IAA at 35 DAP and IAA at 14 DAP had a greater impact on grain-filling parameters. However, IAA at 35 DAP had an advantage in affecting grain-filling parameters in 2020. CTK affected grain-filling parameters at the late grain-filling stage (28 DAP in 2019 and 35 DAP in 2020).

**TABLE 6:** Canonical correlation coefficient between grain-filling parameters and hormone content and standardized analytical model.

Hormone	2019			2020			Canonical variable
	Canonical correlation coefficient	*F*	*P*	Canonical correlation coefficient	*F*	*P*	
IAA	0.974	5.518	0.000	0.965	2.634	0.003	U_2019_ = −0.042a_1_ + 0.276a_2_ + 0.236a_3_ + 0.315a_4_ + 0.388a_5_ (U_2019_ = 0.285a_1_ −1.153a_2_ + 0.503a_3_−0.590a_4_ + 0.805a_5_) U_2020_ = −1.370a_1_ + 0.291a_2_−0.462a_3_ + 0.320a_4_ + 0.291a_5_
	0.875	2.773	0.005	0.635	0.698	0.778	
	0.733	1.680	0.135	0.480	0.437	0.905	
	0.350	0.645	0.635	0.145	0.075	0.989	
	0.210	0.694	0.418	0.016	0.004	0.951	
CTK	0.972	3.390	0.000	0.959	2.634	0.003	U_2019_ = −0.255a_1_−0.007a_2_ + 0.111a_3_−0.196a_4_−0.726a_5_ U_2020_ = −0.225a_1_ + 0.098a_2_−0.508a_3_−0.900a_4_ + 0.095a_5_
	0.711	1.102	0.388	0.712	0.870	0.605	
	0.509	0.766	0.647	0.401	0.403	0.924	
	0.394	0.653	0.630	0.289	0.313	0.867	
	0.097	0.142	0.712	0.014	0.003	0.958	
ABA	0.947	1.833	0.040	0.956	2.606	0.003	U_2019_ = −0.449a_1_ + 0.248a_2_−0.185a_3_−0.057a_4_−0.597a_5_ U_2020_ = −0.251a_1_ + 0.177a_2_ + 0.001a_3_−0.110a_4_−0.816a_5_
	0.517	0.372	0.982	0.729	0.918	0.557	
	0.347	0.209	0.991	0.418	0.393	0.930	
	0.102	0.042	0.996	0.220	0.230	0.919	
	0.039	0.023	0.881	0.121	0.225	0.642	
GA	0.941	2.424	0.005	0.933	2.380	0.006	U_2019_ = 0.355a_1_−0.216a_2_−0.177a_3_−0.743a_4_−0.176a_5_ U_2020_ = 0.459a_1_−0.565a_2_ + 0.342a_3_−1.109a_4_−0.066a_5_
	0.670	1.049	0.433	0.710	1.134	0.362	
	0.543	0.878	0.555	0.588	0.820	0.602	
	0.375	0.701	0.598	0.276	0.300	0.875	
	0.195	0.594	0.453	0.070	0.075	0.788	

*IAA, indole-3-acetic acid; CTK, cytokinin; ABA, abscisic acid; GA, gibberellic acid.*

## Discussion

### Contribution of Yield Components to Yield Under Different Nitrogen Applications

N2 and N3 significantly increased about 200% yield compared to N0, this may N enhance ears ha^–1^, 100-grain weight, and grains per plant, which were the momentous factor influencing maize yield (Wei et al., 2017; Srivastava et al., 2018). Qian et al. (2016) showed that N increased grains per ear and 1,000-grain weight, which subsequently led to an increase in the grain yield. Our result also confirms that yield increased mainly caused by greater ears ha^–1^, associated with an increase in grains per plant, and greater 100-grain weight. The correlations between yield and 100-grain weight (*R*^2^ = 0.514 in 2019, *R*^2^ = 0.602 in 2020) were less than and that yield and ears ha^–1^ (*R*^2^ = 0.836 in 2019, *R*^2^ = 0.645 in 2020), which provided further evidence that grain weight is more conservative than the grains per plant in response to varying environmental conditions (Tsimba et al., 2013). However, once the density and variety are determined, it is difficult to change the ears ha^–1^ unless there is insufficient nutrition (Zhang et al., 2014; Li et al., 2017). As mentioned above, this may also be the reason why there is no significant difference between N2 and N3 on ears ha^–1^, and N0 significantly reduces ears ha^–1^. It is feasible that N optimized maize grain filling increased 100-grain weight since maximum grain size was defined primarily during the lag phase of grain filling (Tsimba et al., 2013). The yield of N3 and N2 did not have a significant difference; this might be because N2 can basically meet fundamental conditions for growing maize in this area, furthermore, N3 promoted the development of vegetative organs but possibly inhibited the transport of nutrients (Li et al., 2017). As reported by Chen et al. (2015), the yield increased obviously with an increase in N application (0–240 kg N ha^–1^); however, enhancing N application has little effect on yield when N application reaches a certain limit. Wazir and Akmal (2019) found that three-split N application maintained ample nutrients for maize grain development, which might profit for higher grain yield. In our study, under the same N rate, N application time could not alter yield significantly. The reason may be due to the fact that plastic film mulch enhanced mineralization of soil N and improves the availability of N, thus weakening the effect of N application time on yield. The yield in T2 was higher than T1 under N3 in 3 years, there may be a more favorable N combination between N2 and N3; however, the specific N application needs further study. The results illustrated that N rate rather than N application time was the central factor affecting the yield when N application rate is less than 200 kg N ha^–1^ (Li and Li, 2015). The yield in 2018, 2019, and 2020 varied wildly. According to Lu et al. (2017), less sunshine inhibits pollination and dry matter accumulation, resulting in lower yield. Precipitation is also a determining factor of yield in rainfed agriculture. Lower yield in 2018 may be due to the lowest in crop rainfall and fewer sunny days during grain filling in 2018.

### Nitrogen Affect Grains Weight by Regulating Grain Filling

Grain filling is an essential physiological process that determines grain formation. Appropriate N rate and time contribute to the rate and duration of grain filling, determining grain weight (Zhou et al., 2017; Wei et al., 2019). In our study, *G*_max_, *G*_max_, *G*_1_, *G*_2_, and *G*_3_ were positively correlated with 100-grain weight, but the *T*_1_ was negatively correlated with 100-grain weight. So, in this area, the filling rate determined the formation of grains under different N rates and time combinations. However, Bolaños (1995) showed that variation in grains weight due to the duration of grain filling but not the grain-filling rate. Similar studies have found that the change in grain weight of the same variety is mainly affected by the filling rate, but the difference of different varieties depends on the duration of grain filling (Yang et al., 2008; Wei et al., 2019). *W*_max_, *G*_max_, *G*_max_, and the duration and average grain-filling rate of GIP, RIP, and SIP improved with the N increase, but N2 and N3 had no significant differences, indicating that N application is not the critical factor to control the filling rate when it reaches 200 kg ha^–1^ in this region. The grain-filling process of maize show an “S”-shaped curve, and the grain-filling rate show the characteristics of slow-fast-slow. In our research, the duration of RIP under different treatments accounted for 27.65∼31.75% of the whole grain-filling duration. Miao et al. (2018) found that the duration of RIP is up to 36.9% of the whole grain-filling duration, so we should pay attention to the duration of RIP to obtain a higher grain weight. There is a mechanism that shortens the duration of GIP while prolonging the period of RIP, increasing 100-grain weight (Wei et al., 2019). Our result also showed that compared to N0, N2, and N3 shorten the duration of the GIP and prolonged the period of RIP, thus increasing grains weight. Under the same N rate, N application time could not alter grain-filling parameters significantly, which determined that the change of grain weight at N application time was not significant.

### Nitrogen Affect Grain Filling Through Regulating Starch Accumulation

Sucrose, the main form of carbon transport, is produced by plant photosynthesis, and the breakdown of sucrose provides intermediates for the synthesis of starch (Farrar et al., 2000). The sucrose content of N2 and N3 was higher than N0 and N1 and reached a maximum value at 14 DAP (except for N2T1 in 2020). These results indicated that N2 and N3 were conducive to sucrose accumulation at the early grain filling, which laid the foundation for starch metabolizing. The reason for sucrose content accumulation is that N increased SPS and SuSy activities, which are the key enzyme in sucrose synthesis (Yang et al., 2008). At 35 DAP, the sucrose content did not show significant changes among N0, N1, N2, and N3, this may be N deficiency preventing the transformation from sucrose to starch (Rufty et al., 1983; Zhao et al., 2013). AGPase and SSS were sensitive to N, indicating that N could regulate the biosynthesis rate of maize starch by affecting AGPase and SSS (Liu et al., 2021). Our result also showed that N2 and N3 could significantly increase AGPase and SSS activities compared to N0. Higher AGPase and SSS activities promoted the accumulation of starch, which is beneficial to the final grain weight (Liang et al., 2017). However, Fahy et al. (2018) found that final grain weight was not determined by AGPase or starch synthase activities during grain filling, and the improvement of grain weight was mainly due to developmental processes before grain filling. Under the same N rate, the starch content and starch metabolizing enzymes showed no significant difference between T1 and T2. This may be one of the reasons why there is no significant change in 100-grain weight between T1 and T2.

### Relationship Between Hormone Content and Grain-Filling Characteristics

Phytohormones, namely, CTK, IAA, ABA, and GA, are instrumental in regulating the development of maize grains by affecting grain cell division and expansion (Yang et al., 2006; Liu et al., 2013). At the grain-filling stage, N application significantly altered hormone contents; however, the change trends (IAA, ABA, and CTK contents first increased and then decreased, GA content declined gradually) were not affected. The proper amount of auxin content can facilitate grain filling by regulating endosperm development, while low IAA constrained the grain weight (Wang et al., 2006). In this study, CTK increased in the early stage of the maize grain-filling period. The high levels of CTKs at the early stage of grains development promote cell division and grain filling (Yang et al., 2000). Our data showed that N deficiency limits the synthesis of CTK, thus reducing grain weight. Liang et al. (2017) found that CTK contents were significantly and positively correlated with the SSS and AGPase activities in grain, higher CTK increased SSS and AGPase activities, thus promoting grain filling. In addition, IAA and CTK content was positively correlated with the *G*_max_ and *G*_max_, and the reason for this may be high IAA levels induced by CTK level (Liu et al., 2016). In our study, the IAA and CTK transiently increased, reached a maximum value at 21 DAP, and then decreased. The canonical correlation between grain-filling parameters and the content of IAA and CTK showed that N application altered the grain-filling process mainly by altering IAA and CTK content. So, the lower 100-grain weight by N deficiency could be attributed to the low CTK and IAA content. ABA has a role in the transport and accumulation of photosynthetic products that regulate the starch-metabolizing enzyme activities in starch biosynthesis (Travaglia et al., 2007; Zhang et al., 2017). The N2 and N3 had significantly increased the ABA content. Previous research showed that the high ABA in the late grain-filling stage was not conducive to the grain filling, and eventually affected grain weight (Wei et al., 2019), and the findings were confirmed as identified by the standardized analytical model in our study. GA promotes endosperm cell proliferation at the early grain-filling progress, but GA increased α-amylase or other hydrolytic enzymes at the late grain filling, which accelerate starch decomposition (Yang et al., 2001; Xu et al., 2015). In this study, GA content reached the maximum value at 7 DAP and increased with increasing N rate, which heightened grain elongation rapidly, and the results are similar with Li et al. (2019). Furthermore, the result also was proved by the standardized analytical model in our study.

## Conclusion

Yield, yield components, grain-filling parameters, starch metabolizing enzymes, and endogenous hormones of maize grain were significantly affected by N rate (200 and 300 kg N ha^–1^ did not have significant difference), but N application time did not show significant difference under the same N rate. As N increased to 200 kg N ha^–1^, the IAA, CTK, and GA content and starch-metabolizing enzymes such as SuSy, SPS, AGPase, and SSS, which regulated grain filling, were significantly improved as compared with 0 kg N ha^–1^. Furthermore, all hormones had strong canonical correlations with the grain-filling parameters (*W*_max_, *G*_max_, *G*_max_, *W*_2_, and *G*_2_). The grain-filling parameters were significantly positively correlated with 100-grain weight. N increased ears ha^–1^, 100-grain weight, and the number of grains per plant, which enhanced maize production compared with 0 kg N ha^–1^. Therefore, we concluded that 200 and 300 kg N ha^–1^ optimized grain-filling parameters by affecting hormones and enzymes, thus improving yield. Considering inputs and environmental factors comprehensively, 200 kg N ha^–1^ with one-third applied at sowing and two-thirds at the six-leaf stage, may be prioritized to choose in the semiarid Loess Plateau of China.

## Data Availability Statement

The raw data supporting the conclusions of this article will be made available by the authors, without undue reservation.

## Author Contributions

KY and LL: conceptualization. KY and JuX: methodology. SF: software. KY, LL, and YL: validation. KY: formal analysis, investigation, and writing—original draft preparation. JuX: resources. KY and JiX: data curation. KY, LL, SA, and SF: writing—review and editing. All authors have read and agreed to the published version of the manuscript.

## Conflict of Interest

The authors declare that the research was conducted in the absence of any commercial or financial relationships that could be construed as a potential conflict of interest.

## Publisher’s Note

All claims expressed in this article are solely those of the authors and do not necessarily represent those of their affiliated organizations, or those of the publisher, the editors and the reviewers. Any product that may be evaluated in this article, or claim that may be made by its manufacturer, is not guaranteed or endorsed by the publisher.
