# Erratum: Nitrogen Supply Affects Yield and Grain Filling of Maize by Regulating Starch Metabolizing Enzyme Activities and Endogenous Hormone Contents

**DOI:** 10.3389/fpls.2022.882425

**Published:** 2022-03-22

**Authors:** Frontiers Production Office

**Affiliations:** Frontiers Media SA, Lausanne, Switzerland

**Keywords:** nitrogen application, maize yield, hormone, starch metabolizing enzymes, grain filling

Due to a production error, the minus sign (–) between “maximal grain” and “filling rate” (equation 2) should be a hyphen (-).

A correction has been made to the section “**Measurements and Calculations for Grain Filling, Enzymes, and Endogenous Hormones Measurements**,” subsection “**Grain-Filling Traits**,” Equation 2:

“Time for maximal grain-filling rate (T_max_) = lnB/C”

Due to a production error, the minus sign (–) between “weight increment” and “achieving maximum” (in equation 3) should be deleted; additionally the minus sign (–) between “Maximum grain” and “filling rate” (equation 2) should be a hyphen (-).

A correction has been made to the section “**Measurements and Calculations for Grain Filling, Enzymes, and Endogenous Hormones Measurements**,” subsection “**Grain-Filling Traits**,” Equation 3:

“Grain weight increment achieving maximum grain-filling rate (W_max_) = A/2”

Due to a production error, the minus sign (–) between “maximum grain” and “filling rate” (equation 4) should be a hyphen (-).

A correction has been made to the section “**Measurements and Calculations for Grain Filling, Enzymes, and Endogenous Hormones Measurements**,” subsection “**Grain-Filling Traits**,” Equation 4:

“Maximum grain-filling rate (G_max_)”

Due to a production error the minus sign (–) between “Active grain” and “filling period” (in equation 5) should be deleted.

A correction has been made to the section “**Measurements and Calculations for Grain Filling, Enzymes, and Endogenous Hormones Measurements**,” subsection “**Grain-Filling Traits**,” Equation 5:

“Active grain-filling period (AGP) = 6/C”

Due to a production error the minus sign (–) between “Average grain” and “filling rate” (in equation 6) should be deleted.

A correction has been made to the section “**Measurements and Calculations for Grain Filling, Enzymes, and Endogenous Hormones Measurements**,” subsection “**Grain-Filling Traits**,” Equation 6:

“Average grain-filling rate (G_max_) = W/56”

The publisher apologizes for these mistakes. The original version of this article has been updated.

